# Acometimento do Ventrículo Direito na Cardiomiopatia por Depósito de Glicogênio (PRKAG2): Análise Ecocardiográfica Convencional e Avançada

**DOI:** 10.36660/abc.20210801

**Published:** 2022-11-11

**Authors:** José Luiz Barros Pena, Fabricio Junqueira de Melo, Wander Costa Santos, Isabel Cristina Gomes Moura, Gabriela Pansanato Nakashima, Natalia Costa Freitas, Eduardo Back Sternick

**Affiliations:** 1 Faculdade de Ciências Médicas de Minas Gerais Belo Horizonte MG Brasil Faculdade de Ciências Médicas de Minas Gerais – Pós-Graduação, Belo Horizonte, MG – Brasil; 2 Hospital Felicio Rocho Belo Horizonte MG Brasil Hospital Felicio Rocho – Ecocardiografia, Belo Horizonte, MG – Brasil

**Keywords:** Síndrome PRKAG2/genética, Doença de Depósito de Glicogênico/complicações, Hipertrofia Ventricular Direita, Cardiomiopatia Hipertrófica Familiar, Ecocardiografia/métodos, Marca-Passo Artificial, Volume Sistólico

## Abstract

**Fundamento:**

A síndrome do PRKAG2 é uma doença hereditária autossômica dominante rara, de início precoce. Objetivamos descrever os achados ecocardiográficos do ventrículo direito (VD) usando modalidades bi e tridimensionais (2D e 3D), incluindo índices de deformação miocárdica nesta cardiomiopatia. Também objetivamos demonstrar se esta técnica poderia identificar alterações na função do VD que pudessem distinguir quaisquer achados particulares.

**Métodos:**

Trinta pacientes com síndrome do PRKAG2 (R302Q e H401Q) geneticamente comprovada, 16 (53,3%) do sexo masculino, com idade média de 39,1 ± 15,4 anos, foram submetidos a exame ecocardiográfico completo. A visão de 4 câmaras com foco no VD foi adquirida para medições 2D e 3D. Os testes t de Student ou Wilcoxon-Mann-Whitney foram usados para comparar as variáveis numéricas entre 2 grupos, e p < 0,05 foi considerado significativo.

**Resultados:**

Doze pacientes (40%) tiveram marca-passo implantado por 12,4 ± 9,9 anos. A espessura diastólica média da parede livre do VD foi de 7,9 ± 2,9 mm. O *strain* longitudinal de 4 câmaras do VD (SL4VD), incluindo a parede livre e o septo interventricular, foi de -17,3% ± 6,7%, e o *strain* longitudinal da parede livre do VD (SLPLVD) foi de −19,1% ± 8,5%. A razão apical do SLPLVD mediu 0,63 ± 0,15. A fração de ejeção (FE) 3D média do VD foi de 42,6% ± 10,9% e abaixo dos limites normais em 56,7% dos pacientes. Correlação positiva ocorreu entre FE 3D do VD, SL4VD e SLPLVD, principalmente para pacientes sem marca-passo (p = 0,006).

**Conclusão:**

O envolvimento do VD em PRKAG2 é frequente e ocorre em diferentes graus. A ecocardiografia é uma ferramenta valiosa na detecção de anormalidades miocárdicas do VD nesta condição. O uso de SL4VD 2D, SLPLVD e FE 3D oferecem indicadores confiáveis de disfunção sistólica do VD nesta cardiomiopatia rara e desafiadora.

## Introdução

O gene PRKAG2 foi inicialmente descrito em 2000 como parte ativa do metabolismo no processo de transcrição da proteína quinase ativada por AMP (AMPK).^1 , 2^ Em quase metade dos casos relatados, as alterações genômicas envolvendo esse gene são devidas à mutação Arg302Gln, que substitui a arginina por glutamina no códon 302, conhecido como R302Q. A literatura também descreve 28 mutações adicionais.^3^ A mutação PRKAG2 resulta na perda da função da subunidade γ2 da AMPK e apresenta um defeito metabólico responsável pela glicogenose. O principal fenótipo consiste em hipertrofia ventricular associada a anormalidades no sistema de condução cardíaca, incluindo síndrome de pré-excitação ventricular.^4^

A mutação PRKAG2 é considerada uma doença rara, embora esteja provavelmente subestimada, pois muitos casos são diagnosticados inadequadamente, sendo muitas vezes classificados como cardiomiopatia hipertrófica familiar. O padrão de herança é dominante, com penetrância completa e graus variados de expressão e prevalência ainda não mencionados na literatura.^5 , 6^

A ecocardiografia bi e tridimensional (2D e 3D) e os índices de deformação miocárdica ( *strain* / *strain rate* ) por *speckle tracking* (STE) são técnicas relativamente recentes, porém já utilizadas para a avaliação da função do ventrículo esquerdo (VE). Mais recentemente, essas técnicas também foram validadas para avaliação da função do ventrículo direito (VD).^7 , 8^

Nosso grupo de pesquisa recentemente publicou um estudo dos achados ecocardiográficos do VE nesta mesma série de pacientes.^9^

A importância reconhecida do VD nas cardiomiopatias está mudando radicalmente, e isso afeta de forma significativa a fisiologia cardíaca, a hemodinâmica e o desenvolvimento de sintomas.^10^ Comparada à circulação sistêmica, a circulação pulmonar apresenta resistência vascular muito menor e maior distensibilidade da artéria pulmonar.^11 - 14^

Objetivamos descrever os achados ecocardiográficos do VD utilizando ecocardiografia 2D e 3D e STE. Também objetivamos identificar se esta técnica poderia eventualmente detectar quaisquer alterações particulares na função do VD na cardiomiopatia por depósito de glicogênio quando comparada ao VE. Visto que existem poucas pesquisas associando os achados ecocardiográficos do VD com a síndrome do PRKAG2, buscamos investigar a presença de parâmetros ecocardiográficos que possam sugerir hipertrofia do VD associada à cardiomiopatia por depósito de glicogênio.

## Métodos

### Pacientes e protocolo de estudo

Trata-se de um estudo observacional, clínico, transversal, baseado em uma coorte de pacientes com síndrome do PRKAG2 geneticamente comprovada. Foram excluídos pacientes com outras etiologias de cardiomiopatia hipertrófica. A população-alvo consistiu em 30 pacientes de 5 famílias com mutação no gene PRKAG2 (28 Arg302Gln e 2 His401Gln), detectados por meio de teste genético de sequenciamento Sanger. Todos os pacientes foram submetidos a exame clínico, com eletrocardiograma convencional de 12 derivações e ecocardiograma. O conselho de revisão institucional aprovou o protocolo e todos os pacientes assinaram um termo de consentimento informado. Nosso estudo foi realizado seguindo as diretrizes das Boas Práticas Clínicas e foi aprovado pelos comitês de ética locais.

### Análise ecocardiográfica

Todos os pacientes foram submetidos a exame ecocardiográfico transtorácico completo, seguindo as recomendações da Sociedade Americana de Ecocardiografia (ASE) e da Associação Europeia de Imagem Cardiovascular (EACVI).^15^ Todos os estudos foram realizados utilizando um sistema ecocardiográfico disponível comercialmente, máquina Vivid E9 (GE Healthcare, Horten, Noruega). O exame incluiu o modo M, medidas 2D, STE 2D de *strain* longitudinal e medidas 3D de acordo com as Diretrizes para a Avaliação Ecocardiográfica do Coração Direito em Adultos: um relatório da ASE.^16^ A visão de 4 câmaras com foco no VD foi adquirida para medidas 2D e 3D, tendo-se o cuidado para obter a imagem que demonstrasse o diâmetro máximo. Foram medidas as dimensões lineares 2D do VD, incluindo as dimensões basais, médias e longitudinais do VD. A via de saída do VD foi medida no final da diástole no corte paraesternal eixo longo. A espessura da parede do VD foi medida na diástole, a partir do corte subcostal, utilizando-se o modo M.

A excursão sistólica do plano anular tricúspide (TAPSE) foi obtida pelo modo M, medida a partir do anel lateral tricúspide.

A veia cava inferior foi medida proximal à junção das veias hepáticas no final da expiração. O *strain* longitudinal de 4 câmaras do VD (SL4VD) foi calculado pela média dos valores de todos os 6 segmentos do VD. O *strain* longitudinal da parede livre do VD (SLPLVD) foi obtida pela média dos 3 segmentos da parede livre do VD: basal, médio e apical. Também calculamos a razão apical da parede livre do VD usando a equação: [strain longitudinal de pico sistólico (SLPS) apical / (SLPS basal + mid-SLPS)]. Todos os dados foram revisados offline. O ecocardiograma transtorácico 3D do VD foi realizado em todos os pacientes. Seis batimentos consecutivos controlados por eletrocardiograma foram adquiridos para gerar o volume completo do VD. O pós-processamento das imagens 3D em tempo real foi realizado no software TomTec 1.1, com o traçado endocárdico de todos os planos. Os volumes do VD foram calculados de forma semiautomática ao longo de todo o ciclo cardíaco, a partir dos quais foram obtidos o volume diastólico final e o volume sistólico final e calculados o volume sistólico e a fração de ejeção (FE). A reprodutibilidade intra e interobservador foi avaliada em uma subamostra de 9 pacientes selecionados aleatoriamente.

### Análise estatística

O tamanho amostral utilizado foi de conveniência devido à raridade dessa condição. As variáveis categóricas foram apresentadas por frequências absolutas e relativas e as variáveis numéricas como média ± desvio padrão se distribuídas normalmente e mediana ± intervalo interquartil se distribuídas anormalmente. A normalidade das variáveis numéricas foi avaliada pelo teste de Shapiro-Wilk. Os testes t de Student ou Wilcoxon-Mann-Whitney foram usados para comparar variáveis numéricas entre 2 grupos usados para amostras independentes. A associação entre as variáveis categóricas foi avaliada pelo teste exato de Fisher. O coeficiente de correlação de Spearman foi utilizado para avaliar a associação entre 2 variáveis numéricas.

Os 30 casos foram atribuídos aleatoriamente números de 1 a 30 usando o software R. Para avaliar a consistência e reprodutibilidade, 2 observadores independentes selecionaram aleatoriamente 9 números para remensuração. A escolha do número de casos foi arbitrária.

As diferenças médias e coeficientes de correlação intraclasse (CCI) foram obtidos. Seus intervalos de confiança (IC) intra e interobservador foram ambos de 95%. As medidas intra e interobservador foram avaliadas pelo teste de Shapiro-Wilk. Testes t de Student para amostras pareadas foram usados para comparar as diferenças médias.

As análises foram realizadas no software R versão 3.5.2, e p < 0,05 foi considerado significativo.

## Resultados

A Tabela 1 mostra as características clínicas e demográficas dos pacientes do estudo. A maioria era do sexo masculino e mais da metade era assintomática. A palpitação foi o sintoma clínico mais frequente. Síndrome de pré-excitação, hipertensão e *flutter* foram os sinais prevalentes.

**Tabela 1:** Características clínicas e demográficas dos pacientes

População, n = 30	
Sexo masculino	16 (53,3%)
Idade (anos)*	39,1 ± 15,2
IMC (kg/m^2^) *	26,9 ± 3,8
ASC (m^2^)*	1,8 ± 0,2
Frequência cardíaca (bpm)**	60,0 (53,0 – 63,0)
**Pressão arterial**	
Sistólica (mmHg)**	120,0 (112,5 – 130,0)
Diastólica (mmHg)**	77,5 (70,0 – 80,0)
**Sinais e sintomas**	
Pré-excitação	19 (63,3%)
Assintomático	16 (53,3%)
Marca-passo	12 (40%)
Hipertensão	10 (33,3%)
Palpitações	7 (23,3%)
Flutter	6 (20%)
Fibrilação atrial	4 (13,3%)
Falta de ar	2 (6,7%)
Pré-síncope	2 (6,7%)

ASC: área de superfície corporal; bpm: batimentos por minuto; IMC: índice de massa corporal. Dados apresentados como * média ± desvio padrão, ** median (1º – 3º quartil).

Os parâmetros ecocardiográficos do VD estão listados na Tabela 2 . A qualidade da imagem 3D foi inadequada em 2 pacientes.

**Tabela 2:** Parâmetros ecocardiográficos do VD de 30 pacientes

Variável	
Espessura da PLVD (mm)**	7,0 (6,0 – 9,0)
TAPSE (mm)*	18,8 ± 3,7
Diâmetro da cavidade basal do VD (mm)*	37,6 ± 5,7
Diâmetro da cavidade média do VD (mm)*	31,0 ± 6,1
Diâmetro longitudinal do VD (mm)**	49,0 (35,0 – 61,0)
Diâmetro da VSVD no PLAX (mm)*	28,0 ± 4,0
VCI no final da expiração (mm)**	17,0 (16,0 – 19,0)
SL4VD (%) **	–18,8 (–14,0 – –20,9)
SLPLVD (%) **	–20,3 (–16,6 – –25,3)
SL basal da PLVD (%) *	–18,0 ± 5,1
SL média da PLVD (%) *	–21,8 ± 5,8
SL apical da PLVD (%) *	–24,3 ± 7,1
Razão apical do VD*	0,63 ± 0,15
VDF 3D do VD (mL) **	95,2 (76,2 – 129,9)
VSF 3D do VD (mL) **	54,0 (44,8 – 69,6)
VS 3D do VD (mL) **	44,6 (30,4 – 59,6)
FE 3D do VD (%) *	42,6 ± 10,9

FE: fração de ejeção; PLAX: corte paraesternal eixo longo; PLVD: parede livre do ventrículo direito; SLPLVD: strain longitudinal da parede livre do ventrículo direito; SL4VD: strain longitudinal de 4 câmaras do ventrículo direito; TAPSE: excursão sistólica do plano anular tricúspide; 3D: tridimensional; VCI: veia cava inferior; VD: ventrículo direito; VDF: volume diastólico final; VS: volume sistólico; VSF: volume sistólico final; VSVD: dia de saída do ventrículo direito. Dados apresentados como * média ± desvio padrão, ** median (1º – 3º quartil).

**Figura 1:**
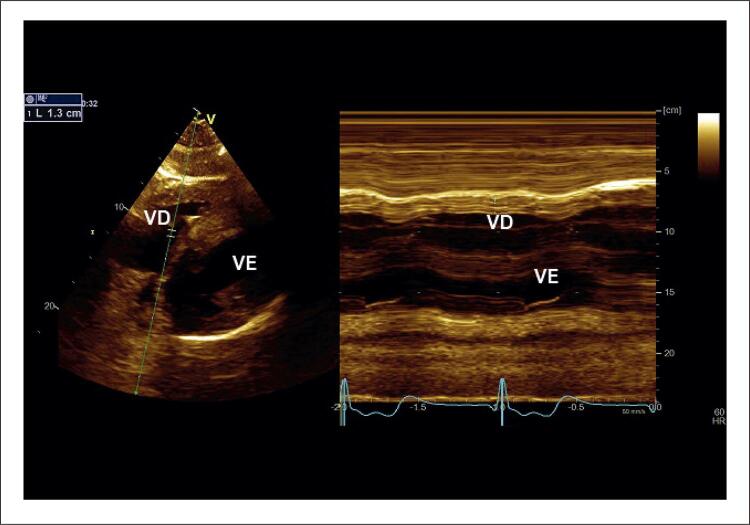
Medida da espessura diastólica final da parede livre do ventrículo direito. Imagem bidimensional subcostal da visão de 4 câmaras. Imagem em modo M indicando a espessura da parede no final da diástole (1,3 cm). VE: ventrículo esquerdo; VD: ventrículo direito.

É importante relatar que, durante o ecocardiograma, apenas 1 paciente apresentou fibrilação atrial. Medida pelo corte subcostal em modo M, a espessura diastólica mediana da parede lateral do VD foi de 7,0 ± 3,0 mm ( Figura 1 ). Apenas 3 pacientes apresentaram valores normais e, em 1 paciente, a medida chegou a 20 mm. Apenas 3 pacientes apresentaram valores de TAPSE abaixo de 17 mm.

A porção anterior do eixo longo paraesternal da dimensão da via de saída do VD no nível proximal apresentou valores superiores ao normal em 23% dos pacientes, conforme relatado na literatura, exceto para a dimensão longitudinal. Esse valor sugere que o aumento da câmara do VD ocorreu na seção transversal.

A regurgitação tricúspide foi detectada em metade dos pacientes, mas apenas 4 apresentaram pressão sistólica da artéria pulmonar acima dos limites normais, e o valor máximo estimado atingiu 48 mmHg.

A veia cava inferior estava dilatada em apenas 2 pacientes.

**Figura 2:**
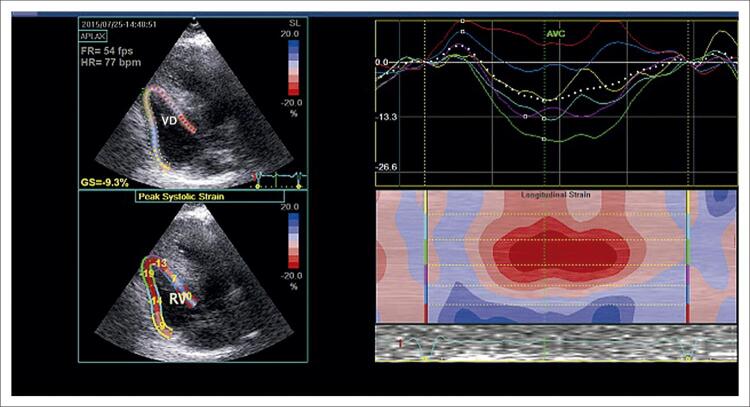
Análise bidimensional do speckle tracking do ventrículo direito a partir de uma visão de 4 câmaras em corte apical. Os valores médios de strain sistólico global e as curvas de tempo foram obtidos pelo rastreamento de uma região de interesse de 6 segmentos. Strain longitudinal de 4 câmaras do ventrículo direito mediu -9,3%. GS: strain global; VD: ventrículo direito.

Em 3 pacientes, o SL4VD estava significativamente reduzido, relacionado à parede lateral mais espessada ( Figura 2 ).

A Tabela 2 também mostra os valores médios de SLPLVD de cada segmento. Podemos observar que os valores basais de SLPLVD são inferiores aos segmentos medial (p = 0,016) e apical (p < 0.001).

**Figura 3:**
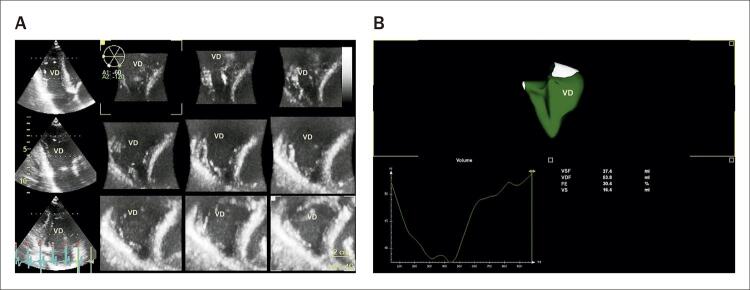
O conjunto de dados tridimensionais foi adquirido a partir de uma visão apical de 4 câmaras com foco no VD. Em A, visão multiplanar em eixo curto para verificação das bordas endocárdicas. Em B, podemos ver o modelo 3D do VD obtido com a curva de volume. VDF: volume diastólico final; FE: fração de ejeção; VSF: volume sistólico final; VD: ventrículo direito; VS: volume sistólico.

A FE do VD estava dentro dos limites normais em 13 pacientes e abaixo de 35% em 7 pacientes ( Figura 3 ).

Os pacientes com marca-passo eram significativamente mais velhos (p < 0,001), e apresentavam maior proporção de fibrilação atrial em comparação aos pacientes sem marca-passo (p = 0,018). O marca-passo foi implantado aos 38,1 ± 13 anos, e o tempo mediano de uso foi de 12,4 ± 9,9 anos.

Os pacientes com marca-passo apresentaram valores significativamente menores de FEVE 3D, encurtamento fracionado e strain circunferencial global 3D.

Verificamos uma diferença estatisticamente significativa entre as medidas do segmento basal e médio da SLPLVD entre os pacientes com e sem marca-passo, conforme mostrado na Tabela 3 .

**Tabela 3:** Parâmetros ecocardiográficos do VD de pacientes sem e com marca-passo

Variável	Sem MP (n=18)	Com MP (n=12)	Valor p
Espessura da PLVD (mm)**	7,0 (6,0 – 8,8)	8,0 (6,5 – 9,0)	0,233 ^W^
TAPSE (mm)*	19,9 ± 2,9	17,0 ± 4,1	0,060 ^T^
Diâmetro da cavidade basal do VD (mm)*	36,8 ± 4,7	39,0 ± 7,1	0,372 ^T^
Diâmetro da cavidade média do VD (mm)*	31,0 ± 5,4	31,1 ± 7,5	0,973 ^T^
Diâmetro longitudinal do VD (mm)*	49,8 ± 13,4	49,0 ± 15,5	0,892 ^T^
Diâmetro da VSVD no PLAX (mm)*	27,0 ± 4,2	29,6 ± 3,3	0,088 ^T^
VCI no final da expiração (mm)**	17,0 (16,0 – 18,8)	18,0 (16,5 – 19,5)	0,440 ^W^
SL4VD (%) *	–18,5 ± 6,8	–15,4 ± 6,4	0,233 ^T^
SLPLVD (%) **	–24,0 (-18,3 – –25,7)	–18,6 (-13,0 – –22,2)	0,187 ^W^
SL basal da PLVD (%) *	–19,7 ± 4,9	–15,6 ± 4,5	0,037^T^
SL média da PLVD (%) **	–26,0 (–18,5 – –26,5)	–19,0 (–14,5 – –23,5)	0,039 ^W^
SL apical da PLVD (%) *	–25,8 ± 7,3	–22,2 ± 6,6	0,200 ^T^
Razão apical do VD*	0,61 ± 0,18	0,65 ± 0,11	0,458 ^T^
VDF 3D do VD (mL)**	95,7 (84,9 – 119,0)	92,9 (69,2 – 149,5)	0,746 ^W^
VSF 3D do VD (mL)**	56,0 (45,7 – 68,4)	51,5 (43,4 – 79,8)	0,963 ^W^
VS 3D do VD (mL)**	45,1 (36,2 – 59,2)	30,6 (29,3 – 59,4)	0,742 ^W^
FE 3D do VD (%) **	48,5 (36,7 – 51,6)	37,5 (32,8 – 40,6)	0,259 ^W^

FE: fração de ejeção; MP: marca-passo; PLAX: corte paraesternal eixo longo; PLVD: parede livre do ventrículo direito; SLPLVD: strain longitudinal da parede livre do ventrículo direito; SL4VD: strain longitudinal de 4 câmaras do ventrículo direito; TAPSE: excursão sistólica do plano anular tricúspide; 3D: tridimensional; VCI: veia cava inferior; VD: ventrículo direito; VDF: volume diastólico final; VS: volume sistólico; VSF: volume sistólico final; VSVD: dia de saída do ventrículo direito. Dados apresentados como * média ± desvio padrão, ** median (1º – 3º quartil). ^*T*^ Teste t de Student t e ^*W*^ teste de Wilcoxon-Mann-Whitney para amostras independentes.

No entanto, não apareceram outras diferenças significativas entre os achados ecocardiográficos do VD entre os pacientes com e sem marca-passo.

As correlações entre as variáveis de *strain* e FE do VD foram avaliadas, conforme apresentado na Tabela 4 . Ocorreu uma correlação positiva entre FE e SLPLVD (r = 0,65, p = 0,006), indicando que quanto maior o valor absoluto de SLPLVD, maiores os valores de EF. Considerando o SL4VD, a correlação foi menor em todos os pacientes e ausente nos pacientes com marca-passo.

**Tabela 4:** Correlações entre FE 3D do VD e SL4VD e SLPLVD em todos os pacientes e naqueles sem e com marca-passo

Grupo	Variável	FE 3D VD	Valor p
Todos pacientes	SL4VD	r = 0,445	0,018
	SLPLVD	r = 0,594	0,001
Sem MP	SL4VD	r = 0,475	0,054
	SLPLVD	r = 0,654	0,006
Com MP	SL4VD	r = 0,355	0,286
	SLPLVD	r = 0,533	0,091

*FE: fração de ejeção; MP: marca-passo; r: coeficiente de correlação de Spearman; SLPLVD: strain longitudinal da parede livre do ventrículo direito; SL4VD: strain longitudinal de 4 câmaras do ventrículo direito; 3D: tridimensional; VD: ventrículo direito.*

Também verificamos uma correlação positiva entre a redução da FEVE e SLPLVD (menos de 50% e −18%, respectivamente) (r = 0,80, p = 0,05).

A reprodutibilidade do *strain* e das medidas de 3D, bem como a CCI e o IC para variabilidade inter e intraobservador estão resumidos na Tabela 5 .

**Tabela 5:** Variabilidade dos dados intra e interobservador

	Intraobservador		Interobservador	

	Média* (95% IC)	CCI (95% IC)	Média* (95% IC)	CCI (95% IC)
SL4VD	0,4 (0,6; 1,5) ^NS^	0,99 (0,93; 1,00) ^†^	1,0 (2,0; 0,7) ^NS^	0,98 (0,74; 1,00)^†^
SLPLVD	0,1 (2,4; 2,6) ^NS^	0,94 (0,65; 0,99)^†^	0,4 (1,9; 2,7) ^NS^	0,96 (0,75; 0,99)^†^
FE 3D	1,3 (1,2; 3,9) ^NS^	0,94 (0,66; 0,99)^†^	0,3 (2,5; 3,2) ^NS^	0,93 (0,61; 0,99)^†^

*Média das diferenças entre as medidas intraobservador (primeira e segunda medidas) e interobservador (observador 1 e observador 2 [dados do estudo coletados]). CCI: coeficientes de correlação intraclasse; FE: fração de ejeção; IC: intervalo de confiança; SLPLVD: strain longitudinal da parede livre do ventrículo direito; SL4VD: strain longitudinal de 4 câmaras do ventrículo direito; 3D: tridimensional. †Valor P < 0,05; NS Valor P ≥ 0,05. Todas as diferenças intra e interobservador apresentaram distribuição normal, conforme avaliada pelo teste de Shapiro-Wilk.

## Discussão

Mutações no gene PRKAG2 alteram a homeostase da AMPK, e a avaliação ecocardiográfica de pacientes com a mutação é uma oportunidade para avaliar as potenciais consequências sistêmicas a longo prazo da ativação da AMPK. Ao avaliar essas consequências, novas linhas de pesquisa podem indicar vias metabólicas envolvidas na fisiopatologia da doença levando ao reconhecimento parcial ou total do fenótipo.^17^ A síndrome do PRKAG2 possui diferentes fenótipos cardíacos, que variam desde uma condição assintomática até a morte súbita cardíaca, incluindo hipertrofia biventricular, pré-excitação, anormalidades na condução atrioventricular, *flutter* atrial e fibrilação.^18 , 19^

Uma grande coorte multicêntrica da Europa foi publicada recentemente relatando dados de 90 pacientes com variantes de PRKAG2.^20^ Este estudo mostrou que pacientes com variantes genéticas de PRKAG2 apresentam prognóstico desfavorável, com alta taxa de complicações, incluindo início juvenil de doença de condução, IC avançada e arritmias potencialmente letais.

A avaliação do tamanho do VD e do desempenho sistólico é cada vez mais solicitada devido ao seu significado reconhecível e prognóstico, especialmente na cardiomiopatia hipertrófica, cardiomiopatia arritmogênica do VD e amiloidose.^21^ Até onde sabemos, esta pesquisa representa o maior estudo ecocardiográfico do VD em uma população com mutação PRKAG2. Visamos descrever os achados do VD nessa rara doença genética e a ocorrência, incidência e grau de disfunção.

O VD foi acometido na grande maioria dos pacientes. A hipertrofia do VD ocorreu em 90% dos pacientes, apresentou padrão regular, envolveu todas as porções da câmara e atingiu 20 mm em 1 caso. Esse achado é semelhante a outras doenças infiltrativas ou genéticas.^22 , 23^ Rosca et al.,^23^ relataram que pacientes com cardiomiopatia hipertrófica apresentaram aumento da espessura da parede do VD em comparação com controles, com aumento do risco calculado de morte súbita cardíaca.^23^

Avaliamos a deformação miocárdica (SL4VD e SLPLVD), volumes do VD e FE desses pacientes. Verificamos que o SLPLVD dos segmentos basais apresentou valores inferiores aos dos segmentos médio e apical. Entretanto, a razão da parede livre do VD mostrou que as análises de *strain* do VD não apresentaram padrão de *apical sparing* , conforme descrito na amiloidose cardíaca sistêmica de cadeia leve.^24^ Vale ressaltar que o SLPLVD foi viável em todos os pacientes.

Verificamos que a FE do VD estava abaixo dos limites normais em mais da metade dos pacientes (56,7%) e, em 7 pacientes, a FE do VD estava abaixo de 35%. Esses valores não foram afetados pelo marca-passo, podendo indicar um sinal diferencial desta doença quando comparado a outros fenótipos hipertróficos. Consideramos FE do VD ≥ 45% como normal.^8^ Alguns pacientes (17,2%) também apresentavam FEVE reduzida, principalmente aqueles com marca-passo. Conforme relatado anteriormente, pacientes com marca-passo apresentaram valores significativamente mais baixos de FEVE 3D, encurtamento fracionado e *strain* circunferencial global 3D.^25^

Uma redução da FE do VD ocorreu em uma proporção maior de pacientes e provavelmente será um sinal diferencial em comparação com outras cardiomiopatias hipertróficas, como as doenças de Fabry e Danon.

A ecocardiografia é uma técnica prática e não invasiva para identificar alterações morfológicas e funcionais na prática clínica.^15^

Mesmo pacientes assintomáticos apresentaram SL4VD e SLPLVD abaixo dos limites normais de referência. Como a viabilidade da estimativa 3D do volume do VD tem sido comprovada nessa síndrome, esse método pode ser aplicado de forma confiável em diagnósticos clínicos.^26 , 27^

A ecocardiografia não apresenta efeitos nocivos em pacientes com marca-passo e tem menor custo, maior portabilidade e maior facilidade de reaplicação do que a ressonância magnética cardíaca.^28^

Observamos que os índices ecocardiográficos convencionais, como TAPSE, não eram indicadores confiáveis para detecção de disfunção do VD. Em estudos anteriores, com outras doenças infiltrativas, esses indicadores mostraram menor sensibilidade para detectar alterações miocárdicas funcionais do que as análises de STE 2D do VD.^27^ É interessante notar que, com o uso do Doppler, não detectamos obstrução na via de saída do VD em repouso. Um relato de caso recentemente publicado detectou uma obstrução dinâmica da via de saída biventricular em um paciente com episódio de síncope. Testes genéticos revelaram que o paciente era heterozigoto para mutação *missense* R302Q no gene PRKAG2, como na maioria dos nossos casos.^26^

Confirmamos uma correlação positiva entre o SLPLVD e a FE do VD, com significância estatística. Esses achados indicam que os índices de deformação são um método rápido e amplamente disponível para detectar disfunção, comparável à FE 3D em pacientes com a mutação PRKAG2. Além disso, ocorreu uma correlação positiva, associando reduções tanto da FEVE quanto da SLPLVD.

Reconhecemos limitações no estudo, como um número relativamente pequeno de pacientes. O software para obtenção de SL4VD e SLPLVD foi adaptado do software desenvolvido para medir o VE. A regurgitação tricúspide foi detectada em metade da população estudada e o aumento da pressão pulmonar sistólica ocorreu em 4 pacientes, que foi avaliada exclusivamente por esse método.

São recomendadas pesquisas adicionais usando esses critérios prospectivamente e o uso de diferentes técnicas de imagem para comparação para validar ainda mais nossos achados.

## Conclusão

O envolvimento do VD em PRKAG2 é frequente e ocorre em diferentes graus. A ecocardiografia é uma ferramenta valiosa na detecção de anormalidades miocárdicas do VD na cardiomiopatia PRKAG2. SL4VD 2D, SLPLVD e FE 3D são indicadores confiáveis de disfunção sistólica do VD nesta doença rara. Estudos longitudinais adicionais são necessários para melhor entender a história natural do envolvimento do VD e determinar seu impacto nos desfechos dos pacientes.
