# Supplementary material for: Acometimento do Ventrículo Direito na Cardiomiopatia por Depósito de Glicogênio (PRKAG2): Análise Ecocardiográfica Convencional e Avançada
Source: Arq Bras Cardiol. 2022 Nov 11;119(6):902–9. [Article in Portuguese] doi: 10.36660/abc.20210801 (PMC9814818; doi:10.36660/abc.20210801)
Supplement: Translation

# Translation: Right Ventricle Involvement by Glycogen Storage Cardiomyopathy (PRKAG2): Standard and Advanced Echocardiography Analyses

**Authors:** José Luiz Barros Pena, Fabricio Junqueira de Melo, Wander Costa Santos, Isabel Cristina Gomes Moura, Gabriela Pansanato Nakashima, Natalia Costa Freitas, Eduardo Back Sternick

**Affiliations:** 1 Brazil Faculdade de Ciências Médicas de Minas Gerais – Pós-Graduação, Belo Horizonte, MG – Brazil; 2 Brazil Hospital Felicio Rocho – Ecocardiografia, Belo Horizonte, MG – Brazil

**Keywords:** PRKAG2 Syndrome/genetics, Glycogen Storage Disease/complications, Hypertrophy, Right Ventricular, Cardiomyopathy, Hypertrophic Familial, Echocardiography/methods, Pacemaker, Artificial, Stroke Volume

## Abstract

**Background:**

PRKAG2 syndrome is a rare, early-onset autosomal dominant inherited disease. We aimed to describe the right ventricle (RV) echocardiographic findings using two and three-dimensional (2D and 3D) modalities including myocardial deformation indices in this cardiomyopathy. We also aimed to demonstrate whether this technique could identify changes in RV function that could distinguish any particular findings.

**Methods:**

Thirty patients with genetically proven PRKAG2 (R302Q and H401Q), 16 (53.3%) males, mean age 39.1 ± 15.4 years, underwent complete echocardiography examination. RV-focused, 4-chamber view was acquired for 2D and 3D measurements. Student’s t or Wilcoxon-Mann-Whitney tests were used to compare numerical variables between 2 groups, and p < 0.05 was considered significant.

**Results:**

Twelve patients (40%) had a pacemaker implanted for 12.4 ± 9.9 years. RV free wall mean diastolic thickness was 7.9 ± 2.9 mm. RV 4-chamber longitudinal strain (RV4LS), including the free wall and interventricular septum, was –17.3% ± 6.7%, and RV free wall longitudinal strain (RVFWLS) was −19.1% ± 8.5%. The RVFWLS apical ratio measured 0.63 ± 0.15. Mean RV 3D ejection fraction (EF) was 42.6% ± 10.9% and below normal limits in 56.7% of patients. Positive correlation occurred between RV 3DEF, RV4LS, and RVFWLS, especially for patients without a pacemaker (p = 0.006).

**Conclusion:**

RV involvement in PRKAG2 syndrome is frequent, occurring in different degrees. Echocardiography is a valuable tool in detecting RV myocardial abnormalities in this condition. The use of 2D RV4LS, RVFWLS, and 3DEF offers reliable indicators of RV systolic dysfunction in this rare, challenging cardiomyopathy.

## Introduction

The PRKAG2 gene was initially described in 2000 as an active part of metabolism in the transcription process of AMP-activated protein kinase AMPK).^1 , 2^ In nearly half of the reported cases, genomic changes involving this gene are due to the Arg302Gln mutation, which replaces arginine with glutamine at codon 302, known as R302Q. The literature also describes 28 additional mutations.^3^ The PRKAG2 mutation results in loss of function of the γ2 subunit of AMPK and features a metabolic defect responsible for glycogen metabolic disease. The main phenotype consists of ventricular hypertrophy associated with abnormalities in the cardiac conduction system, including ventricular pre-excitation syndrome.^4^

PRKAG2 mutation is considered a rare disease, although it is probably underestimated, because many cases are improperly diagnosed, often being labeled as familial sarcomeric hypertrophic cardiomyopathy. The inheritance pattern is dominant, with complete penetrance and varying degrees of expression and prevalence still unmentioned in the literature.^5 , 6^

Two- and three-dimensional (2D and 3D) echocardiography and myocardial deformation indices (strain/strain rate) by speckle tracking (STE) are relatively recent techniques, yet they are already used for assessing left ventricle (LV) function. More recently, these techniques have also been validated for assessing right ventricle (RV) function.^7 , 8^

Our research group recently published a study of the LV echocardiography findings in this same series of patients.^9^

The recognized importance of the RV in cardiomyopathies is radically changing, and this significantly affects cardiac physiology, hemodynamics, and the development of symptoms.^10^ Compared to systemic circulation, pulmonary circulation has a much lower vascular resistance and greater pulmonary artery distensibility.^11 - 14^

We aim to describe the RV echocardiographic findings using 2D and 3D echocardiography and STE. We also aim to identify if this technique could eventually detect any particular changes in RV function in glycogen storage cardiomyopathy when compared to LV. As little research exists associating RV echocardiographic findings with PRKAG2 syndrome, we seek to investigate the presence of echocardiographic parameters that could suggest RV hypertrophy associated with glycogen deposit cardiomyopathy.

## Methods

### Patients and study protocol

This is an observational, clinical, transversal study, based on a cohort of patients with genetically proven PRKAG2 syndrome. Patients with other hypertrophic cardiomyopathy etiologies were excluded. The target population consisted of 30 patients from 5 families with PRKAG2 gene mutation (28 Arg302Gln and 2 His401Gln), detected utilizing Sanger sequential genetic testing. All patients underwent clinical examination, with a standard 12-lead electrocardiogram and echocardiogram. The institutional review board approved the protocol and all patients signed a written informed consent form. Our study was performed following the guidelines of Good Clinical Practice and was approved by the local ethics committees.

### Echocardiographic analysis

All patients underwent a complete transthoracic echocardiography examination, following the recommendations of the American Society of Echocardiography (ASE) and the European Association of Cardiovascular Imaging (EACVI).^15^ All studies were performed using a commercially available echocardiographic system, Vivid E9 machine (GE Healthcare, Horten, Norway). The examination included M-mode, 2D measurements, 2D STE of longitudinal strain, and 3D measurements according to The Guidelines for the Echocardiographic Assessment of the Right Heart in Adults: a report from the ASE.^16^ The RV-focused, 4-chamber view was acquired for 2D and 3D measurements, and care was taken to obtain the image demonstrating the maximum diameter. RV 2D linear dimensions were measured, including RV basal, mid-cavity, and longitudinal dimensions. The RV outflow tract was measured at end-diastole in the parasternal long-axis view. RV wall thickness was measured in diastole, from the subcostal view, using M-mode imaging.

Tricuspid annular plane systolic excursion (TAPSE) was obtained by M-mode, measured from the tricuspid lateral annulus.

Inferior vena cava was measured proximal to the junction of the hepatic veins at end-expiration. RV 4-chamber longitudinal strain (RV4LS) was calculated by averaging values of all 6 RV segments. RV free wall longitudinal strain (RVFWLS) was obtained by averaging the 3 free wall RV segments: basal, mid, and apical. We also calculated the RVFW apical ratio using the equation: [apical peak systolic longitudinal strain (PSLS) / (basal PSLS + mid-PSLS)]. All data were reviewed offline. RV transthoracic 3D echocardiography was performed in all patients. Six electrocardiogram-gated consecutive beats were acquired to generate the full RV volume. Post-processing of real-time 3D images was performed using TomTec software 1.1, with the endocardial tracing of all planes. RV volumes were semi-automatically computed throughout the entire cardiac cycle, from which end-diastolic volume and end-systolic volume were obtained, and stroke volume and ejection fraction (EF) were calculated. Intra- and interobserver reproducibility were assessed on a subsample of 9 randomly selected patients.

### Statistical analysis

The sample sized used was a convenience sample due to the rarity of this condition. Categorical variables were presented by absolute and relative frequencies and numerical variables as mean ± standard deviation if normally distributed and median ± interquartile range if abnormally distributed. The normality of the numerical variables was assessed using the Shapiro-Wilk test. Student’s t or Wilcoxon-Mann-Whitney tests were used to compare numerical variables between 2 groups used for independent samples. The association between categorical variables was assessed using Fisher’s exact test. Spearman’s correlation coefficient was used to assess the association between 2 numerical variables.

The 30 cases were randomly assigned numbers from 1 to 30 using R software. To assess consistency and reproducibility, 2 independent observers randomly selected 9 numbers for remeasurement. The choice for the number of cases was arbitrary.

Mean differences and intraclass correlation coefficients (ICC) were obtained. Their intra- and interobserver confidence intervals (CI) were both 95%. Intra- and interobserver measurements were assessed using the Shapiro-Wilk test. Student t-tests for paired samples were used to compare mean differences.

The analyses were performed using R software version 3.5.2, and p < 0.05 was considered significant.

## Results

Table 1 shows the clinical and demographic characteristics of the patients in the study. The majority were male, and more than half were asymptomatic. Palpitation was the most frequent clinical symptom. Pre-excitation syndrome, hypertension, and flutter were prevalent signs.

**Table 1:** Clinical and demographic characteristics of patients

Population, n = 30	
Male	16 (53.3%)
Age (years)*	39.1 ± 15.2
BMI (kg/m^2^) *	26.9 ± 3.8
BSA (m^2^)*	1.8 ± 0.2
Heart rate (bpm)**	60.0 (53.0 – 63.0)
**Blood pressure**	
Systolic (mmHg)**	120.0 (112.5 – 130.0)
Diastolic (mmHg)**	77.5 (70.0 – 80.0)
**Signs and symptoms**	
Pre-excitation	19 (63.3%)
Asymptomatic	16 (53.3%)
Pacemaker	12 (40%)
Hypertension	10 (33.3%)
Palpitations	7 (23.3%)
Flutter	6 (20%)
AF	4 (13.3%)
Shortness of breath	2 (6.7%)
Presyncope	2 (6.7%)

AF: atrial fibrillation; BMI: body mass index; BSA: body surface area: bpm: beats per minute. Data showed as * mean ± standard deviation, ** median (1st – 3rd quartile).

RV echocardiographic parameters are listed in Table 2 . The 3D image quality was inadequate in 2 patients.

**Table 2:** Echocardiographic RV parameters of 30 patients

Variable	
RVFW thickness (mm)**	7.0 (6.0 – 9.0)
TAPSE (mm)*	18.8 ± 3.7
RV basal cavity diameter (mm)*	37.6 ± 5.7
RV mid cavity diameter (mm)*	31.0 ± 6.1
RV longitudinal diameter (mm)**	49.0 (35.0 – 61.0)
RVOT PLAX diameter (mm)*	28.0 ± 4.0
IVC at end-expiration (mm)**	17.0 (16.0 – 19.0)
RV4LS (%) **	–18,8 (–14,0 – 20,9)
RVFWLS (%) **	–20.3 (–16.6 – 25.3)
RVFW basal LS (%) *	–18.0 ± 5.1
RVFW mid LS (%) *	–21.8 ± 5.8
RVFW apical LS (%) *	–24.3 ± 7.1
RV apical ratio*	0.63 ± 0.15
RV 3D EDV (mL) **	95.2 (76.2 – 129.9)
RV 3D ESV (mL) **	54.0 (44.8 – 69.6)
RV 3D SV (mL) **	44.6 (30.4 – 59.6)
RV 3D EF (%) *	42.6 ± 10.9

EDV: end-diastolic volume; EF: ejection fraction; ESV: end-systolic volume; IVC: inferior vena cava; PLAX: parasternal long-axis; RV: right ventricle; RV4LS: right ventricle longitudinal strain; RVFW: right ventricle free wall; RVFWLS: right ventricle free wall longitudinal strain; RVOT: right ventricular outflow tract; SV: stroke volume; TAPSE: tricuspid annular plane systolic excursion; 3D: three-dimensional. Data showed as * mean ± standard deviation, ** median (1st – 3rd quartile).

It is important to report that, during the echocardiogram, only 1 patient presented atrial fibrillation. Measured by the subcostal view using M-mode, RV lateral wall median diastolic thickness was 7.0 ± 3.0 mm ( Figure 1 ). Only 3 patients presented normal values, and, in 1 patient, the measurement reached 20 mm. Only 3 patients showed TAPSE values below 17 mm.

**Figure 1:** Measurement of end-diastolic right ventricle free wall thickness. Subcostal 2-dimensional image of the 4-chamber view. M-mode image indicating wall thickness at end-diastole (1.3 cm). LV: left ventricle; RV: right ventricle.

Parasternal long-axis anterior portion of RV outflow tract dimension at the proximal level presented higher values compared to normal in 23% of patients, as reported in the literature, except for the longitudinal dimension. This value suggests that the RV chamber increase occurred in the transverse section.

Tricuspid regurgitation was detected in half of patients, but only 4 presented pulmonary artery systolic pressure above normal limits, and the maximum estimated value reached 48 mmHg.

Inferior vena cava was dilated in only 2 patients.

In 3 patients, RV4LS was significantly reduced, relating to more thickened lateral wall ( Figure 2 ).

**Figure 2:** Two-dimensional speckle tracking analysis of the right ventricle from a focused apical 4-chamber view. The global average systolic strain values and time curves are obtained by tracking of a 6-segment region of interest. Right ventricle 4-chamber longitudinal strain measured 9.3%. GS: global strain; RV: right ventricle.

Table 2 also shows the mean RVFWLS values of each segment. We can observe that the RVFWLS basal values are lower than medial (p = 0.016) and apical segments (p < 0.001).

RVEF was within normal limits in 13 patients and below 35% in 7 patients ( Figure 3 ).

**Figure 3:** The 3-dimensional dataset was acquired from an RV-focused apical 4-chamber view. In A, there is a multiplanar short-axis view to verify the endocardial borders. In B, we can see the RV 3-dimensional model obtained with the volume curve. EDV: end diastolic volume; EF: ejection fraction; ESV: end systolic volume; RV: right ventricle; SV: stroke volume.

Patients with a pacemaker (PM) were significantly older (p < 0.001), and they had a higher proportion of atrial fibrillation compared to patients without PM (p = 0.018). The pacemaker was implanted at 38.1 ± 13 years, and the median time of use was 12.4 ± 9.9 years.

Patients with PM presented significantly lower values of 3D LVEF, fractional shortening, and 3D global circumferential strain.

We detected a statistically significant difference between measurements of RVFWLS basal and mid-segment between patients with and without PM, as shown in Table 3 .

**Table 3:** Echocardiographic RV parameters of patients without and with pacemaker

Variable	Without PM (n=18)	With PM (n=12)	p-value
RVFW thickness (mm)**	7.0 (6.0 – 8.8)	8.0 (6.5 – 9.0)	0.233^W^
TAPSE (mm)*	19.9 ± 2.9	17.0 ± 4.1	0.060^T^
RV basal cavity diameter (mm)*	36.8 ± 4.7	39.0 ± 7.1	0.372^T^
RV mid cavity diameter (mm)*	31.0 ± 5.4	31.1 ± 7.5	0.973^T^
RV longitudinal diameter (mm)*	49.8 ± 13.4	49.0 ± 15.5	0.892^T^
RVOT PLAX diameter (mm)*	27.0 ± 4.2	29.6 ± 3.3	0.088^T^
IVC at end-expiration (mm)**	17.0 (16.0 – 18.8)	18.0 (16.5 – 19.5)	0.440^W^
RV4LS (%) *	–18.5 ± 6.8	–13.0 ± 6.4	0.233^T^
RVFWLS (%) **	–24.0 (–18.3 – –25.7)	–18.6 (–13.0 – 22.2)	0.187^W^
RVFW basal LS (%) *	–19.7 ± 4.9	–15.6 ± 4.5	0.037^T^
RVFW mid LS (%) **	–26.0 (-18.5 – –26.5)	–19.0 (–14.5 – –23.5)	0.039^W^
RVFW apical LS (%) *	–25.8 ± 7.3	–22.2 ± 6.6	0.200^T^
RV apical ratio*	0.61 ± 0.18	0.65 ± 0.11	0.458^T^
RV 3D EDV (mL)**	95.7 (84.9 – 119.0)	92.9 (69.2 – 149.5)	0.746^W^
RV 3D ESV (mL)**	56.0 (45.7 – 68.4)	51.5 (43.4 – 79.8)	0.963^W^
RV 3D SV (mL)**	45.1 (36.2 – 59.2)	30.6 (29.3 – 59.4)	0.742^W^
RV 3D EF (%) **	48.5 (36.7 – 51.6)	37.5 (32.8 – 40.6)	0.259^W^

EDV: end-diastolic volume; EF: ejection fraction; ESV: end-systolic volume; IVC: inferior vena cava; PLAX: parasternal long-axis; PM: pacemaker; RV: right ventricle; RV4LS: right ventricle longitudinal strain; RVFW: right ventricle free wall; RVFWLS: right ventricle free wall longitudinal strain; RVOT: right ventricular outflow tract; SV: stroke volume; TAPSE: tricuspid annular plane systolic excursion; 3D: three-dimensional. Data showed as * mean ± standard deviation, ** median (1st – 3rd quartile). T Student’s t and W Wilcoxon-Mann-Whitney test for independent samples.

However, no other significant differences appeared among the RV echocardiographic findings between patients with and without PM.

The correlations between the strain variables and RVEF were assessed, as shown in Table 4 . A positive correlation occurred between EF and RVFWLS (r = 0.65, p = 0.006), indicating that the higher the absolute value of RVFWLS, the higher the EF values. Considering RV4LS, correlation was lower in all patients and absent in patients with PM.

**Table 4:** Correlations between 3D RV EF and RV4LS and RVFWLS in all patients and those without and with a pacemaker

Group	Variable	3D RV EF	p-value
All patients	RV4LS	r = 0.445	0.018
	RVFWLS	r = 0.594	0.001
Without PM	RV4LS	r = 0.475	0.054
	RVFWLS	r = 0.654	0.006
With PM	RV4LS	r = 0.355	0.286
	RVFWLS	r = 0.533	0.091

*EF: ejection fraction; PM: pacemaker; r: Spearman’s correlation coefficient; RV: right ventricle; RV4LS: right ventricle longitudinal strain; RVFWLS: right ventricle free wall longitudinal strain; 3D: three-dimensional.*

We also found a positive correlation between the reduction of LVEF and RVFWLS (less than 50% and −18%, respectively) (r = 0.80, p = 0.05).

The reproducibility of strain and 3D measurements, and the ICC and CI for inter- and intraobserver variability are summarized in Table 5 .

**Table 5:** Intra- and interobserver data variability

	Intraobserver		Interobserver	

	Mean* (95% CI)	ICC (95% CI)	Mean* (95% CI)	ICC (95% CI)
RV4LS	0.4 (0.6; 1.5)^NS^	0.99 (0.93; 1.00)^†^	1.0 (2.0; 0.7)^NS^	0.98 (0.74; 1.00)^†^
RVFWLS	0.1 (2.4; 2.6)^NS^	0.94 (0.65; 0.99)^†^	0.4 (1.9; 2.7)^NS^	0.96 (0.75; 0.99)^†^
3D EF	1.3 (1.2; 3.9)^NS^	0.94 (0.66; 0.99)^†^	0.3 (2.5; 3.2)^NS^	0.93 (0.61; 0.99)^†^

*Mean of differences between intraobserver measurements (first and second measurements) and interobserver (observer 1 and observer 2 [collected study data]). CI: confidence interval; EF: ejection fraction; ICC: intraclass correlation coefficient; RV4LS: right ventricle longitudinal strain; RVFWLS: right ventricle free wall longitudinal strain; 3D: three-dimensional.^*†*^ P-value < 0.05; NS P-value ≥ 0.05. All intra- and interobserver differences showed normal distribution, as assessed by the Shapiro-Wilk test.

## Discussion

Mutations in the PRKAG2 gene alter AMPK homeostasis, and the echocardiographic assessment of patients with the mutation is an opportunity to assess the potential long-term systemic consequences of AMPK activation. By assessing these consequences, new lines of research could indicate metabolic pathways involved in the disease pathophysiology leading to partial or total phenotype recognition.^17^ PRKAG2 syndrome has different cardiac phenotypes, ranging from an asymptomatic condition to sudden cardiac death, including biventricular hypertrophy, pre-excitation, atrioventricular conduction abnormalities, atrial flutter, and fibrillation.^18 , 19^

A large multicenter European cohort was recently published reporting data from 90 patients with PRKAG2 variants.^20^ This study showed that patients with PRKAG2 genetic variants have a poor prognosis with a high rate of complications, including juvenile onset of conduction disease, advanced HF, and potentially lethal arrhythmias.

Evaluation of RV size and systolic performance is increasingly in demand due to its recognizable significance and prognosis, especially in hypertrophic cardiomyopathy, arrhythmogenic RV cardiomyopathy, and amyloidosis.^21^ To the best of our knowledge, this research represents the largest RV echocardiographic study in a population with PRKAG2 mutation. We aimed to describe the RV findings in this rare genetic disorder and the occurrence of dysfunction, incidence, and degree of dysfunction.

The RV was affected in the great majority of patients. RV hypertrophy occurred in 90% of patients, presented a regular pattern, involved all portions of the chamber, and reached 20 mm in 1 case. This finding is similar to other infiltrative or genetic diseases.^22 , 23^ Rosca et al.^23^ related that patients with hypertrophic cardiomyopathy had increased RV wall thickness compared to controls with increased calculated sudden cardiac death risk.^23^

We evaluated these patients’ myocardial deformation (RV4LS and RVFWLS), RV volumes, and EF. We found that the RVFWLS of basal segments showed lower values than mid and apical segments. However, the RVFW ratio showed that the RV strain analyses presented no apical sparing pattern, as described in systemic light-chain cardiac amyloidosis.^24^ It is noteworthy that RVFWLS was feasible in all patients.

We found that RVEF was below normal limits in more than half of patients (56.7%), and, in 7 patients, RVEF was below 35%. These values were unaffected by PM, and they could indicate a differential signal of this disease when compared to other hypertrophic phenotypes. We considered normal RVEF ≥ 45%.^8^ Some patients (17.2%) also presented reduced LVEF, especially patients with PM. As previously reported, patients with PM presented significantly lower values of 3D LVEF, fractional shortening, and 3D global circumferential strain.^25^

RVEF reduction occurred in a greater proportion of patients and will likely be a differential signal compared to other hypertrophic cardiomyopathies such as Fabry and Danon diseases.

Echocardiography is a practical, non-invasive technique to identify morphological and functional alterations in clinical practice.^15^

Even asymptomatic patients presented RV4LS and RVFWLS below normal reference limits. As the feasibility of 3D RV volume estimation has been proven in this syndrome, this method can be reliably applied in clinical diagnoses.^26 , 27^

Echocardiography presents no harmful effects in patients with PM, and it has lower cost, higher portability, and easier reapplication than cardiac magnetic resonance.^28^

We observed that conventional echocardiographic indices, like TAPSE, were unreliable indicators for RV dysfunction detection. In previous studies, with other infiltrative diseases, these indicators showed less sensitivity to detect functional myocardial alterations than RV 2D STE analyses.^27^ Interestingly, by using Doppler, we detected no obstruction in the RV outflow tract at rest. A recently published case report detected a dynamic biventricular outflow tract obstruction in a patient with a syncopal episode. Genetic testing revealed that the patient was heterozygous for R302Q missense mutation in the PRKAG2 gene, as in the majority of our cases.^26^

We confirmed a positive correlation between RVFWLS and RVEF, with statistical significance. These findings indicate that the deformation indices are a fast and widely available method to detect dysfunction, comparable to 3D EF in patients with the PRKAG2 mutation. Additionally, a positive correlation occurred, associating reductions of both LVEF and RVFWLS.

We recognize limitations in the study, such as a relatively small number of patients. The software for obtaining RV4LS and RVFWLS was adapted from the software designed to measure the LV. Tricuspid regurgitation was detected in half of the study population, and increased systolic pulmonary pressure occurred in 4 patients, which was evaluated solely by this method.

Additional research using these criteria prospectively and the use of different imaging techniques for comparison are recommended to further validate our findings.

## Conclusion

RV involvement in PRKAG2 is frequent and occurs in different degrees. Echocardiography is a valuable tool in detecting RV myocardial abnormalities in PRKAG2 cardiomyopathy. Two-dimensional RV4LS, RVFWLS, and 3D EF are reliable indicators of RV systolic dysfunction in this rare disease. Additional longitudinal studies are warranted to further understand the natural history of RV involvement and determine its impact on patient outcomes.
